# Smartphones in the secondary prevention of cardiovascular disease: a systematic review

**DOI:** 10.1186/s12872-018-0764-x

**Published:** 2018-02-07

**Authors:** Sandra J. Hamilton, Belynda Mills, Eleanor M. Birch, Sandra C. Thompson

**Affiliations:** 1 Western Australian Centre for Rural Health, University of Western Australia, 35 Stirling Highway, Crawley, WA 6009 Australia; 2 Georgetown University, 3700 O St NW, Washington, DC 20057 USA

## Abstract

**Background:**

Cardiac Rehabilitation (CR) and secondary prevention are effective components of evidence-based management for cardiac patients, resulting in improved clinical and behavioural outcomes. Mobile health (mHealth) is a rapidly growing health delivery method that has the potential to enhance CR and heart failure management. We undertook a systematic review to assess the evidence around mHealth interventions for CR and heart failure management for service and patient outcomes, cost effectiveness with a view to how mHealth could be utilized for rural, remote and Indigenous cardiac patients.

**Methods:**

A comprehensive search of databases using key terms was conducted for the years 2000 to August 2016 to identify randomised and non-randomised trials utilizing smartphone functionality and a model of care that included CR and heart failure management. Included studies were assessed for quality and risk of bias and data extraction was undertaken by two independent reviewers.

**Results:**

Nine studies described a mix of mHealth interventions for CR (5 studies) and heart failure (4 studies) in the following categories: feasibility, utility and uptake studies; and randomised controlled trials. Studies showed that mHealth delivery for CR and heart failure management is feasible with high rates of participant engagement, acceptance, usage, and adherence. Moreover, mHealth delivery of CR was as effective as traditional centre-based CR (TCR) with significant improvement in quality of life. Hospital utilization for heart failure patients showed inconsistent reductions. There was limited inclusion of rural participants.

**Conclusion:**

Mobile health delivery has the potential to improve access to CR and heart failure management for patients unable to attend TCR programs. Feasibility testing of culturally appropriate mHealth delivery for CR and heart failure management is required in rural and remote settings with subsequent implementation and evaluation into local health care services.

**Electronic supplementary material:**

The online version of this article (10.1186/s12872-018-0764-x) contains supplementary material, which is available to authorized users.

## Background

Cardiovascular disease (CVD) is a leading cause of morbidity and mortality and the leading disease category for health-care expenditure in Australia [1, 2]. Cardiac Rehabilitation (CR) and secondary prevention are components of evidence-based management assisting patients with CVD (coronary artery disease, heart failure, atrial fibrillation and peripheral artery disease) return to an active and satisfying life through improved clinical and behaviour outcomes and helps reduce the recurrence of cardiac events [3–5].

Cardiac rehabilitation (CR) is a coordinated multidimensional evidence-based strategy that aims to assist patients with CVD return to “an active and satisfying life and to prevent the recurrence of cardiac events” [6]. Secondary prevention, is defined as “healthcare designed to prevent recurrence of cardiovascular events or complications of CVD in patients diagnosed with CVD” [7]. Although these definitions are similar, CR may be time limited, whereas secondary prevention proposes a cardiac rehabilitation continuum where care is provided for the rest of a person’s life according to need [7].

Cardiac rehabilitation is known to be underutilised: in Australia, attendance rates at traditional CR programs are estimated to be as low as 10–30% even in metropolitan areas, with even greater under-representation of rural, remote and Indigenous populations [5, 8]. Low CR attendance rates can reflect factors at the health service and broader system level, and well as health professional and patient related factors. These are significantly greater for people who live in rural and remote settings [8–11]. Systems and health professional related barriers limit accessibility through referral failure [8], absence of local CR programs and limited program places [8], program inflexibility [8, 10, 11], and failure to meet the needs of individual patients [10].

Nearly one third of the Australian population reside in rural and remote areas, and despite similar rates of CVD, their cardiovascular outcomes are poorer than for those living in metropolitan areas [12]. Furthermore, the proportion of Aboriginal and Torres Strait Islander (hereafter Indigenous) Australians, known to have higher rates and earlier onset of CVD, increases with remoteness [13]. This vulnerable population is among those with more prevalent comorbidities who are less likely to receive, adhere to and complete CR [8, 11], with its consequent suboptimal clinical benefit.

The care that patients receive is in part a function of the characteristics of health systems [14]. Inadequate health information systems and communication impede referral processes, service provision and continuity of care and contribute to referral failure, poor uptake and attendance and lower completion of CR for rural, remote and Indigenous patients [15]. For rural and remote patients, program availability and/or inflexibility, geographical location (distance, time and transport difficulties), hours of program scheduling, and cultural inappropriateness reduces accessibility and increases cost [8, 10, 11]. Alternative models of CR (Table 1), including patient-centred telehealth and community- or home-based CR, are preferred by many patients [5, 16–18]. These models encompass eight broad categories and have generally produced similar reductions in CVD risk factors compared with traditional outpatient CR [5].

**Table 1:** Alternative models of cardiac rehabilitation

• multifactorial individualized telehealth delivery providing individualized assessment and risk factor modification with patient-provider contact primarily by telephone;
• internet-based delivery of programs where the majority of patient-provider contact is via the internet;
• exercise telehealth interventions where patient-provider contact is primarily by telephone;
• telehealth interventions focused on psycho-social recovery where patient-provider contact is primarily by telephone;
• community or home-based CR involving patient-provider contact during home visits or patient visits to a community centre;
• program delivery to diverse population groups including rural and remote settings;
• multifaceted models of care incorporating interventions across these categories;
• models utilizing complementary or alternative medicine

Information and communication technologies (ICTs) have increasingly been incorporated into health care systems including innovative CR delivery [19, 20]. ICTs include a variety of applications/ platforms which enable users to access, store, transmit and manipulate information electronically (eHealth). Advances have been enabled by the uptake of mobile technology, with 31 million mobile phone connections for a resident population of 23.6 million (131 mobile phones per 100 citizens in Australia) in June 2014 [21]. The uptake of smartphone technology in Australia has been rapid [22], with 89% of the 2014 Australian Mobile Phone Lifestyle Index survey respondents aged 18–75 years owning a smartphone. Mobile phone use was preferred (50%) compared with a tablet device (16%) or a personal computer (34%). Health and wellbeing information had been accessed by 58% of the survey respondents within the last 12 months, and was used by 15% at least once weekly. Health and wellbeing applications (apps) were utilised by 27% of the survey respondents [22].

Mobile health (mHealth), a component of eHealth, is a rapidly growing health delivery methodology with the potential to impact on health care research, health care delivery and health outcomes [23]. Specifically, mHealth has the potential to enhance primary and secondary disease prevention and deliver interventions that are personalized, adaptive and sustainable, improve patient communication, access to health care services and treatment, and patient engagement and provide real-time medication monitoring and adherence support [23]. However, smartphone interventions may be limited by cost, especially for people with lower socioeconomic status who may have limited ability to pay the cost of receiving extra data via their smartphone. Furthermore, rural and remote populations may have poor access to data connectivity which limits smartphone use.

mHealth broadly refers to medical and public health practice supported by mobile devices [24] and includes mobile phones, smartphones, patient monitoring devices, personal digital assistants (PDAs) and other wireless devices. Mobile and smartphones provide the functionality of voice and short messaging service (SMS and/or text messaging), while smartphone functionality allows for downloaded programs (apps), numerous interfaces and specialized capabilities including third and fourth generation mobile telecommunications (3G and 4G systems), global positioning system (GPS) and Bluetooth connectivity [24, 25]. mHealth enable consumers or providers to monitor health status through wireless diagnostic and clinical decision support [26]. The widespread adoption of smartphones and their integral role in people’s lifestyle as a communication tool make them an attractive platform for the accurate capture of measurements and delivery of flexible health interventions or programs such as CR [20].

This systematic review examines smartphone interventions for comprehensive CR and heart failure rehabilitation/management for service and patient outcomes and how they can be utilised for cardiac patients in rural and remote settings.

## Methods

### Search strategy

A comprehensive search of electronic databases utilizing key terms relating to the research question was undertaken for the years 2000 to August 2016. The database search was supplemented by a manual search (pearling) of reference lists of included studies. Eligible studies were published in peer reviewed journals and in English. Randomised and non-randomised studies (randomised control trial (RCT)), quasi-experimental, or observational) with a prospective experimental study design (quantitative and qualitative) utilizing smartphone functionality and a model of care that included CR and/or secondary cardiovascular prevention and heart failure rehabilitation were eligible for inclusion. Studies were excluded if they were retrospective, non-intervention studies, systematic reviews, study protocols, conference abstracts and papers reporting on content or technical development. We excluded studies which were primarily text messaging or web-based interventions.

The following databases were searched: PubMed, Medline*,* Academic Search Premier, CINAHL Plus, Embase, Google Scholar, and Cochrane Library. Boolean operators were utilized to combine key terms and MeSH terms including: rural, remote, regional, indigenous, cardiac rehabilitation, secondary cardiovascular prevention, healthcare applications, mHealth, eHealth, mobile, smartphone, computer, tele*, internet, web*, technolog*, communication, applications*, alternative methods, home-based (Additional file 1).

### Study selection

Two reviewers independently screened potential articles for inclusion. The PRISMA guide (Preferred reporting items for systematic reviews and meta-analyses) was followed for study inclusion. Duplicate publications were removed, then titles and abstracts were screened for relevance. Full-texts of the remaining publications were retrieved by two reviewers and assessed against the inclusion and exclusion criteria (Table 2).

**Table 2:** Inclusion and exclusion criteria

P	Patients eligible for Cardiac Rehabilitation and Secondary Prevention (acute coronary syndrome, re-vascularisation procedures, controlled heart failure and other vascular or heart disease [6, 59]) or heart failure management.
I	A model of care that utilized smartphone functionality (either app or Wireless Application Protocol (WAP) capabilities) for comprehensive Cardiac Rehabilitation and Secondary Prevention or heart failure rehabilitation
C	None, traditional cardiac rehabilitation or usual care
O	Feasibility, utility, and uptake of mHealth; service outcomes (patient engagement, acceptance, adherence and completion, provider engagement and acceptance, and cost effectiveness); patient outcomes (clinical, exercise capacity, knowledge, social and emotional, QOL); health service utilisation.
E	Retrospective studies; non-intervention studies; systematic reviews; study protocols; conference abstracts and non-cardiac rehabilitation or heart failure programs.

Key**:** *P* Patient, *I* Intervention, *C* Comparison intervention, *O* Outcomes, *QOL* Quality of life, *E* Exclusion criteria

### Data extraction and analysis

Studies initially considered suitable were reviewed by two independent reviewers using a template based on the Joanna Briggs Institute (JBI) Reviewers guidelines [27]. Publications were grouped by cardiac rehabilitation and heart failure and key information on study design, model of care, intervention and methods were synthesized.

Assessment of the level of evidence utilised the National Health and Medical Research Council’s (NHMRC) Evidence Hierarchy [28]. Two reviewers also appraised the included studies for quality and risk of bias utilizing the Critical Appraisal Skills Programme (CASP) tools for methodological rigour of study design and the quality in reporting [29].

## Results

**Fig. 1:**
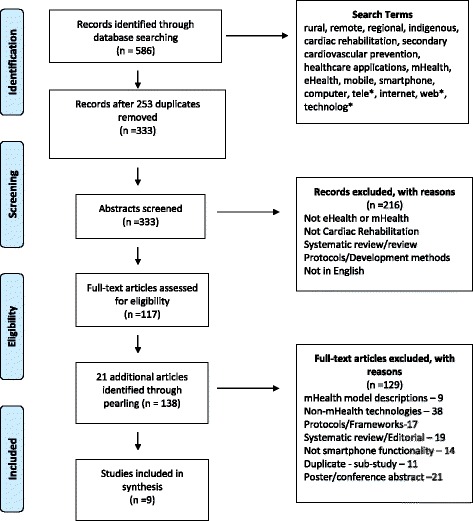
Flow Diagram of Search Results

The initial search identified 586 records, with an additional 21 records identified through a manual search of reference lists of included studies (pearling). Following removal of duplicates, abstract and full-title assessment, nine articles were considered eligible and included in the review (Fig. 1).

The included articles described a mix of mHealth interventions for CR (Tables 3, 4 and 5) and heart failure (Tables 6, 7 and 8) of two key types: (1) Feasibility, utility and uptake (FUU) studies: observational studies focussing on the feasibility and/or utility of the intervention and reporting on participant uptake and acceptance; (2) RCTs: single-blind or open-label RCTs which compared a mHealth intervention with traditional CR (TCR) or usual heart failure management alone.

**Table 3:** Levels of evidence and outcome measures in cardiac rehabilitation studies

	Worringham, 2011	Forman, 2014	Varnfield, 2011	Blasco, 2012	Varnfield, 2014
Country of Origin	Australia	USA	Australia	Spain	Australia
Levels of evidence					
Design	FUU	FUU	FUU	RCT	RCT
Level of Evidence*	IV	IV	IV	II	II
CASP score	n/a	n/a	n/a	7	8
Theoretical framework			✓		✓
Outcome measures					
Technology					
Feasibility	✓	✓	✓	✓	
Usability	✓	✓	✓		
Technical problems	✓		✓		✓
Acceptability		✓	✓	✓	✓
Engagement	✓	✓			
Adherence	✓	✓	✓	✓	✓
Usage			✓		
Task completion		✓			
Patient					
Uptake	✓		✓		✓
PROs		✓			
Program completion	✓				✓
Qualitative feedback	✓	✓	✓		
CV risk improvement				✓	
Physical activity			✓	✓	✓
Step counter			✓		✓
6MWT	✓				✓
Nutrition			✓	✓	✓
Smoking status				✓	
Psychological distress					✓
Depression	✓				✓
Anxiety				✓	✓
QOL	✓			✓	✓
Self-efficacy					
BP and HR			✓	✓	✓
Weight			✓		✓
BMI				✓	✓
Waist circumference					✓
HbA1_c_				✓	✓
Plasma lipid level				✓	✓
PVO_2_					
Medication adherence					
Economic evaluation					✓

Key: ✓ Outcome measured, *FUU* Feasibility, Utility and Uptake study, *RCT* Randomised Control Trial, *n/a* Not applicable, *CASP* Critical Appraisal Skills Programme, *PROs* Patient reported outcomes, *CV* Cardiovascular, *6MWT* Six minute walk test, *QOL* Quality of life, *BP* Blood pressure, HR Heart rate, *BMI* Body mass index, *HbA1*_*c*_ Haemoglobin A1_c_, *PVO*_*2*_ Peak oxygen uptake. *National Health and Medical Research Council's (NHMRC) Evidence Hierarch [28].

**Table 4:** Summary of outcome results in cardiac rehabilitation studies

	Worringham, 2011 (FUU)	Forman, 2014 (FUU)	Varnfield, 2011 (FUU)	Blasco, 2012 (RCT)	Varnfield, 2014 (RCT)
Patient numbers n =	6	26	15	102 TMG; 101CG	60 CAP; 60 TCR
Program completion	100%	NR	Internal feasibility study	87% TMG: 88% (*n* = 90 of 102) CG: 87% (*n* = 88 of 101, includes 4 deaths) or 91% (n 92 of 101, excludes 4 deaths)	++ TMG: 80% (n 48 = of 60) vs CG: 47% (*n* = 28 of 60)
Mean age (years)	53.6 (42–67)	59 (43–76) 33% > 65 years	59	60.6 ± 1.3 TMG 61.0 ± 12.1 CG	54.9 ± 9.6 CAP 56.2 ± 10.1 TCR
Technology (reported inconsistently)					
Feasibility	✓	✓	NR		
Usability/Acceptability	4.8/5.0 Ease of use	83% positive experience	Easy (qualitative data)		
Technical	80% of completed sessions had no technical problems	NR	36% use of Wellness Diary Connected internet -limited by computer and internet access	42% of TMG withdrawals due to technical issues (n = 5 of 12)	7% of CAP withdrawals reported technical difficulties
Engagement/Adherence/Usage/Task completion	87% completed sessions	90% daily engagement 78% task completion	91.5% wellness diary 100% step counter	98% completed > 50% sessions 83% completed > 75% of sessions	++ (94% vs 68% adherence)
Patient					
Uptake	86% of referred patient		Internal feasibility study	83.5%	++ (80% vs 62%)
Mentorship			91% motivational		
CV risk improvement				++ (ITT)	
Physical activity				ns	
6 Minute Walk Test	+				ǂ † ns
Nutrition					ǂ † ns
Smoking cessation				ns (ITT)	
Psychological distress					ǂ ns
Depression	+				ǂ † ns
Anxiety					ǂ ns
Quality of Life	+ − physical health ns - mental health (SF36)			++ Physical health ns Mental health (SF36)	ǂ ++ (EQ5D-Index)
Blood pressure (BP) and Heart rate (HR)				++ BP (ITT) ++ SBP ≠ DBP	++ DBP ns HR ns SBP
Weight					ǂ ns
Body Mass Index				++	
Waist circumference					ǂ ns
Haemoglobin A1_c_ (HbA1_c_)				++ (ITT) ++	ns
Plasma lipid level				ns LDL-c (ITT) ++ LDL-c ++ TG	† ns TC ǂ † ≠ TG ns LDL-c

Key: *FUU* Feasibility, Utility and Uptake study, *RCT* Randomised Control Trial, *TMG* Telemonitoring Group, *CAP* Care Assessment Platform cardiac rehabilitation, *TCR* Traditional Cardiac Rehabilitation, *CG* Control Group, *PROs* Patient reported outcomes, *CV* Cardiovascular, *LDL-c* Low density lipoprotein cholesterol, *TG* Triglyceride

FUU studies - pre- compared with post-intervention: + = significant improvement with mHealth; # = no significant improvement with mHealth; NR = not reported; ✓ = reported as feasible

RCT studies: ++ = significant improvement in mHealth group compared with TCR (or CG) group; ns = no significant difference between mHealth group and TCR (or CG) group; ≠ = significant improvement in TCR (or CG) compared with mHealth group; ǂ = significant CAP within-group differences at 6 weeks; † = significant TCR within-group differences at 6 weeks; ITT = Intention to treat analysis

**Table 5:** Cardiac Rehabilitation Studies

Publication (Author, Year, Country)	Participants, Sample Size, Rurality and Theoretical Model	mHealth and Non-mHealth Components	Intervention and Comparison	Outcomes
Worringham, 2011 Australia	Patients with an acute coronary event or revascularisation procedure unable to attend traditional CR Referred patients: *n* = 7 (1 withdrew prior to initial exercise testing) Intervention: *n* = 6 (83% M) Metropolitan and rural participants Theoretical model: None	*mHealth* Programmed smartphone External heart and activity monitor with GPS and Bluetooth connectivity Real-time monitoring of location, speed, heart rate and single lead ECG. Mobile phone contact pre- and post-exercise sessions Emergency mobile phone contact	Non-randomised feasibility trial Comparison group: None 6 week intervention Long-term engagement: recognised as a limitation and need for larger-scale and longer-term studies. Examination of outcomes of an outdoor walking-based exercise program *Statistical analysis:* Paired *t*-tests.	*Mean age* 53.6 (42–67) years *Uptake:* 86% of referred patients *Completion/attrition:* 100% completed the 6 week exercise program *Usability and adherence:* 87% of sessions completed (80% without technical problems), 13% cancelled. *Ease of use* rated 4.8/5 (95%CI 4.6–5.0) *Physical function:* 6MWT improved from 524 m to 637 m (*p* = 0.009). *SF36 QOL:* Physical Health score increased from 50.0 to 78.4 (*p* = 0.03) *Mental Health improved:* Cardiac depression scale reduced from 54.0 to 44.6 (p- = 0.007). SF36 QOL Mental Health score: NS
Forman, 2014 USA	Patients currently enrolled in Phase 2 CR or recently completed (within 1 month) and continuing with Phase 3 CR. *n* = 26 (77% M) Metropolitan Hospital Rural patients not reported Theoretical model: None	*mHealth* iPhone, iPad or iPod touch Heart Coach Application: • Daily messages and tasks • Educational material and videos • Medication reminders • Physical activity prompts • Screenings and surveys *Non-mHealth* Traditional centre-based phase 2 CR or phase 3 CR (not defined) Qualitative patient and clinician feedback	Observational feasibility and utility study Comparison group: None Exercise and education-based mHealth program to augment traditional centre-based CR effectiveness 30 day intervention period Long-term engagement: no engagement or follow-up reported beyond the 30 day intervention period. Primary outcomes: qualitative feedback and engagement with technology *Statistical analysis:* Data collected and structured automatically by the application and presented as reports. Qualitative data assessed through surveys.	*Mean age* 59 (43–76) years; 33% aged > 65 years *Study completion/attrition:* not reported *Usability (mean):* 90% daily engagement *Utility:* Task completion • 78% overall • 88% educational • 82% survey • 79% medication reminder • 70% physical activity (30 min) on 3 days/week Heart Coach had 42% lower visit cancellations vs no Heart Coach Staff typically spent about 20 min a day reviewing all patients’ progress and sending patient messages. (32 messages per patient over the study) Positive impact and reinforced impact of clinical-based sessions
Varnfield, 2011 Australia	Post-MI patients eligible for CR *n* = 15% M/F not reported Metropolitan CR centres Rural patients not included Theoretical model: Self-management	*mHealth* Smartphone: • Integrated accelerometer sensor • step counter • Wellness Diary • Relaxation audio files • Educational multimedia • Weekly telephone mentoring sessions • Text messages *Non-mHealth* Clinical Review at CR centre (baseline and 6 weeks). Face-to-face training on CAP BP monitor and Weight scales (data entered into Wellness Diary) ‘My Heart My Life’ manual Participant and Mentor questionnaires on usability and uptake of technology and adherence. Internet web portal for viewing of patient data by mentors	Internal feasibility study: preliminary analysis of CAP data from RCT (CAP vs TCR). Comparison group: None Comprehensive delivery of core components of CR: Exercise and education-based intervention 6 week CAP CR program Long-term engagement: not reported *Statistical analysis:* Uptake and use determined through data uploaded daily to remote web portal.	*Patients:* Mean age 59 years *Study completion/attrition:* Internal feasibility study of 15 CAP participants *Uptake, Usability and adherence:* Average usage rate: Wellness Diary 91.5%; Step Counter 97%; Wellness Diary Connected internet application use was 36% due to lack of computer or internet connection; 91% reported that phone contact with mentor was motivational. *Mentors:* *Usability and safety:* CAP practical and easy to use with benefits to patients (reduced travel, return to work). Concerns over lack of exercise supervision, individual motivation levels and group support.
Blasco, 2012 Spain	Patients with ACS and one CV risk factor (tobacco smoking, LDLc ≤100 mg/dl (2.6 mmol/L), hypertension; or diabetes mellitus) *n* = 203 (80% M) TMG: *n* = 102 (30 T2D; 81% M) CG: *n* = 101 (26 T2D; 79% M) Metropolitan tertiary hospital Rural patients not reported. Theoretical model: None	*mHealth* Nokia mobile phone with Wireless Application Protocol (WAP) technology and secure Web Portal *Patient:* BP, HR, weight (weekly), glucose and lipids (monthly) levels sent through mobile phone via structured questionnaire. *Cardiologist:* Secure Web Portal for access of results. Individualized recommendations via short text messages *Non-mHealth* *TMG* Omron automatic blood pressure monitor, CardioChek glucose and lipid meter. Patient Satisfaction Questionnaire at exit visit *All* Baseline and exit visit: clinical assessment, blood samples and SF-36 and State-Trait Anxiety Inventory for adults. 3 clinical visits with cardiologist	Single-blind RCT Telemonitoring Group (TMG) vs Control group (CG). All patients received lifestyle counselling and usual care 12 month follow-up Long-term engagement: no engagement or follow-up reported beyond the 12 month intervention period. *Statistical analysis:* Intention to treat. Independent and paired *t* tests; *X*^2^ test and relative risk	*Mean age* (years) ± SD: 60.6 ± 11.3 (TMG) vs 61.0 ± 12.1 (CG) *Primary Outcome:* CV Risk improvement: TMG (*n* = 87) 69.6% vs CG (*n* = 83) 50.5% *P* = 0.01 TMG vs CG meeting treatment goals for BP < 140/90 mmHg (62.1% vs 42.9%, *p* = 0.012); HbA1_c_ < 7% (86.4% vs 54.2%, *p* = 0.018); smoking cessation (*p* = 0.964) and LDL-C (*p* = 0.948) *Completion/attrition:* 87% (*n* = 177) completion rate with a 13% attrition - 4 participants were lost to follow-up and 5 died (all in the CG). 17 participants left the study (12 TMG and 5 CG): Reasons were stress of telemonitoring (*n* = 3 TMG), personal reasons (*n* = 7 TMG, *n* = 5 CG) and inability to operate equipment (*n* = 2 TMG) *Adherence to protocol:* 98% completed > 50% of sessions; 83% completed > 75% of sessions.
Varnfield, Nov 2014 Australia	Post-MI patients *n* = 94 (82 M; 12 F) CAP: *n* = 53 (91% M) TCR: *n* = 41 (83% M) Metropolitan CR centres Rural patients not included Theoretical model: Self-management	*mHealth* *CAP-CR Patient: Smartphone* • Integrated accelerometer sensor • Step counter • Wellness Diary • Relaxation audio files • Educational multimedia *Community Care Team* • Internet web portal for viewing of patient data • Text messages • Video and Telephone mentoring *Non-mHealth* *All:* ‘My Heart My Life’ manual *CAP-CR* Clinical Review at CR centre. Face-to-face training in CAP. Blood pressure monitor and Weight scales *TCR program* Two supervised exercise and 1 h education sessions weekly for 6 weeks at CR centre.	RCT of CAP compared with TCR. Comprehensive CR program 6 week CR intervention Long-term engagement: included a Self-management phase. CAP-CR participants kept smartphone and monitoring devices for this phase. Participants were encouraged to maintain lifestyle changes. Secondary outcomes, activity monitoring and perception of using a smartphone to monitor exercise was measured at 6 months *Statistical analysis:* ITT basis; Chi squared; Independent *t* test; Wilcoxon rank-sum test; ANCOVA adjusted for age and gender; Linear mixed model regression	*Mean age* (years) ± SD: 54.9 ± 9.6 (CAP) vs 56.2 ± 10.1 (TCR) *Primary outcome:* *Uptake:* 80% CAP vs 62% TCR, *P* < 0.05 *Adherence:* 94% CAP vs 68% TCR, P < 0.05 *Study completion*: 80% CAP vs 47% TCR completed, *p* < 0.05 *Attrition: N* = 44 dropouts, 70% from TCR (non-uptake / non-completion). Life demands: TCR - 10% Work, 4% stress; CAP – 0% Logistics: TCR - 16% Time, 7% location, 2% transport; CAP - 2% time Change in circumstances: TCR - 14% health, 2% criteria; CAP - 9% health, 7% smartphone Study design: TCR - 10%; CAP - 0% Motivation: TCR – 4%; CAP – 2% Improved health: TCR – 0%; CAP – 2% Privacy: TCR – 2%; CAP -)% Other reasons: TCR – 2%; CAP – 5% *Technology:* 7% (*n* = 3) reported difficulty with mHealth tools *Secondary outcomes:* CAP was as effective as TCR in improving: dietary intake; depression; 6MWT and triglycerides (*p* < 0.05). CAP effectively reduced psychological distress; anxiety levels; weight, WC and HRQOL (p < 0.05). TCR effectively reduced TC (*P* = 0.04) Between-group differences: DBP and HRQOL < 0.05 for CAP and Tgs < 0.05 for TCR *Cost analysis*: Based on 2010 Australian health economics data, CAP CR may result in AU$16.6 million readmission cost savings

Key: *n/a* not applicable, *CR* Cardiac Rehabilitation, *NS* No Significant Change, *RCT* Randomised Controlled Trial, *MI* Myocardial Infarction, *CAP* Care Assessment Platform, *TCR* Traditional Cardiac Rehabilitation, *T2D* Type 2 Diabetes, *6MWT* 6 Minute Walk Test, *WC* Waist Circumference, *DPB* Diastolic Blood Pressure, *EQ5D-Index* A Health-related Quality of Life Index, *Tgs* Triglycerides, *TC* Total Cholesterol, *ACS* Acute Coronary Syndrome, *CV* Cardiovascular, *TMG* Telemonitoring Group, *IG* Intervention Group, *CG* Control Group, *BP* Blood Pressure, *BMI* Body Mass Index, *LDL-C* Low-density Lipoprotein Cholesterol, *HbA1*_*c*_ Glycated Haemoglobin A1_c_, *SF-36* Health Related Quality of Life Short Form 36, *QOL* Quality of Life, *HC* Heart Coach application, *PVO*_*2*_ peak oxygen uptake, *wk*. week, *ANCOVA* Analysis of Covariance, *NZ* New Zealand; *ns* no significant difference, *SD* Standard Deviation

**Table 6:** Levels of evidence and outcome measures in Heart Failure studies

	Scherr, 2006	Scherr, 2009	Seto, 2012	Vuorinen 214
Country of Origin	Austria	Austria	Canada	Finland
Levels of evidence				
Design	FUU	RCT	RCT	RCT
Level of Evidence*	IV	II	II	II
CASP score**	n/a	6	8	8
Theoretical framework			✓	
Outcome measures				
Technology				
Reliability	✓			
Feasibility	✓			
Clinical utility	✓		✓	
Usability	✓	✓		✓
Acceptability	✓			✓
Adherence	✓	✓	✓	✓
Usage	✓	✓	✓	✓
Task completion	✓			
Patient				
Patient satisfaction	✓			✓
Qualitative feedback			✓	✓
QOL			✓	
Self-care			✓	✓
NYHA class	✓	✓	✓	
LVEF	✓	✓	✓	✓
BP and HR	✓	✓	✓	
Weight	✓	✓	✓	
ECG			✓	
Medication use	✓	✓	✓	
Biochemistry			✓	✓
BNP			✓	✓
Mortality		✓	✓	✓
Health service utilization		✓	✓	✓
Economic evaluation	None of these smartphone heart failure studies included a health economic analysis			

Key: ✓ Outcome measured, *FUU* Feasibility, Utility and Uptake study, *RCT* Randomised Control Trial, *n/a* Not applicable, *CASP* Critical Appraisal Skills Programme, *QOL* Quality of life, *NYHA* New York Heart Association, *LVEF* Left ventricular ejection fraction, *BP* Blood pressure, *HR* Heart rate, *ECG* Electrocardiogram, *BNP* Brain Natriuretic Peptide

**Table 7:** Summary of outcome results in Heart Failure studies

	Scherr, 2006 (FUU)	Scherr, 2009 (RCT)	Seto, 2012 (RCT)	Vuorinen 2014 (RCT)
*n* =	20 14 CHF; 6 HTN	120 66 TMG (54 + 12 never beginners) 54 CG	100 50 TMG; 50 SCG	94 (47 TMG; 47 CG)
Program completion	95% (*n* = 19 of 20) CHF: 93% (*n* = 13 of 14) HTN: 100%	87% (*n* = 104 of 120) TMG: 76% (*n* = 50 of 66, includes never beginners) or 93% (n = 50 of 54) CG: 100%	97% (*n* = 97 of 100) TMG: 88% (*n* = 44 of 47, includes 3 deaths) or 94% (*n* = 47 of 50, excludes 3 deaths) SCG: 100%	99% (*n* = 1 of 94) TMG: 98% (n = 1 of 47) CG: 100%
Mean age (years)	50 (SD14) CHF: 53 (13); HTN: 42 (16)	66 (IQR 64–74) TMG: 66 years (IQR 62–73) CG: 67 years (IQR 61–72)	TMG: 55.1 ± 13.7 SCG: 52.3 ± 13.7	TMG: 58.3 ± 11.6 CG: 57.9 ± 11.9
Technology (Reported inconsistently)				
Feasibility	✓high			
Usability/Acceptability	80% did not report any problems with data entry	98% system availability		10–20 min initial education on use of mobile phone app. TMG: 1 patient withdrew due to increased anxiety from monitoring his condition.
Technical	98% data transmission and website availability One (5%) withdrawl (poor vision)	12 never beginners (median age 68 years (IQR 64–74) were unable to begin transmission of data (reasons NR)	TMG: 2 participants withdrew due to technical difficulties	TMG: 6 telephone calls re technical problems. 3 nurse initiated calls for start-up support; 3 patient calls initiated for failed internet connection.
Engagement/Adherence/ Usage/Task completion	94% (CHF) and 84% (HTN) self-measurement and data entry	95% patient adherence	Completion of daily readings: 84% completed 50%; 66% completed 80%; 32% completed 95%.	Proportion of weekly submitted self-measurements by TMG: 86% weight (median = 28 (IQ 23–33); 89% BP, HR, and symptoms (median BP and symptoms = 32 (IQR 27–43).
Patient				
Patient satisfaction	85% of patients continued telemonitoring at study completion			96% responded to user experience questionnaire 95% - measures very or quite useful 91% - automatic feedback very or quite useful (9% no benefit) 66% - feedback drew attention to essential issues of disease 91% - feedback was motivational
Quality of Life			ǂ overall MLHFQ ǂ Physical ǂ Emotional ++ Overall change (*p* = 0.05)	
Self-care			ǂ † Maintenance ǂ † Management ++ Maintenance	ns ǂ †
New York Heart Association class	Study completion vs baseline Class I: *n* = 3 vs 0 Class II: *n* = 11 vs 10 Class III: *n* = 0 vs 4	++ (PPA)	ǂ †	
Left Ventricular Ejection Fraction	↑ in mean to 35% at study completion (vs 32% at baseline)	PPA: ns improvement TMG: 25% (IQR 20–38) to 35% (IQR 25–45) CG: 29% (IQR 21–36) to 35% (IQR 24–40)	ǂ †	ns ǂ †
Blood Pressure	HTN: mean study completion SBP 135 (SD18); DPB 78 (7) vs baseline SBP 134 (21); DBP 80 (8)			
Medication	CHF: 71% had beta-blocker therapy initiated with a titrated increase HTN: antihypertensive medication stable		ǂ Aldosterone antagonists	++ Medication change, both increases and decreases.
Biochemistry				ns Serum potassium, creatinine, sodium.
Brain Natriuretic Peptide			ǂ †	ns ǂ
Mortality/Health service utilization		ITT: ns TMG: 17% (0 deaths/11) hospitalizations CG: 31% (1 death/17 hospitalizations) PPA: ++ TMG: 15% (0 deaths/8 hospitalizations) PPA: ++ shorter length of hospital stay	TMG: 6% (n = 3) deaths (2 non-heart related) SCG: 0 deaths ns hospital admissions; nights in hospital; ED visits ++ Heart Function Clinic visits (TMG: 3.5 (SD 3.6); SCG 2.5 (2.5)	No mortality in TMG or CG ns HF related hospital days ++ TMG nurse time, telephone contact and visits ++ TMG unplanned clinic visits ++ patient initiated telephone contact ns physician time and visits

Key: *FUU* Feasibility, Utility and Uptake study, *RCT* Randomised Control Trial, CHF Chronic Heart Failure, *HTN* Hypertension, *TMG* Telemonitoring Group, *SCG* Standard Care Group, *CG* Control Group, *QOL* Quality of life, *SBP* Systolic Blood Pressure, *DBP* Diastolic Blood Pressure, ECG Electrocardiogram, *ED* Emergency Department, *NR* not reported

FUU studies - pre- compared with post-intervention: + = significant improvement with mHealth; # = no significant improvement with mHealth; NR = not reported; ↑ = increased; ✓ = reported as feasible

RCT studies: ++ = significant improvement in TMG compared with SCG (or CG); ns = no significant difference between TMG and SCG (or CG);

≠ = significant improvement in SCG (or CG) compared with TMG; ǂ = significant TMG within-group differences at 6 months; † = significant SCG (or CG) within-group differences at 6 months; ITT = Intention to treat analysis: PPA = Per Protocol Analysis

**Table 8:** Heart Failure Studies

Publication (Author, Year, Country)	Participants, Sample Size, Rurality and Theoretical Model	mHealth and Non-mHealth Components	Intervention	Outcomes
Scherr, 2006 Austria	Patients with chronic Heart Failure CHF or hypertension (HTN) *n* = 20 (95% M) CHF: *n* = 14 (93% M) HTN: *n* = 6 (83% M) Metropolitan and rural patients Theoretical model: None Comparison group: None	*mHealth* Patient terminal: Mobile phone with Wireless Application Protocol (WAP) technology Physician’s terminal: Personal Computer with internet access *Non-mHealth* Automatic BP monitor Digital weight scales Doctor–patient relationship Patient completed questionnaires on the technical aspects of the telemonitoring system	Observational study to evaluate acceptability, feasibility and reliability of a telemonitoring system. 90 day follow-up Long-term engagement: no engagement or follow-up reported beyond the 90 day intervention period. Patients measured BP, HR and weight daily and transferred data via mobile phone. Physician automatically notified by SMS of any parameters outside a pre-set range. Study physician accessed data and phoned patient as necessary for therapeutic adjustments Automatic reminders set by study physician *Statistical analysis:* Descriptive statistics reported.	*Mean Age:* All, 50 (SD14) years; CHF, 53 (SD13) years; HTN, 42 (SD 16) years. *Study completion/attrition:* 95% (*n* = 19) completed CHF: 93% (*n* = 13) completed HTN: 100% (*n* = 6 completed) 5% (n = 1) withdrew from TMG due to poor vision *Reliability* 98% data transmission success 98% website availability for physicians. *Feasibility and Acceptability:* Implausible data entry: 5 per CHF patient; 4 per patients with HTN Successful transmissions: 83% CHF and 84% HTN Self-measurement and data entry: CHF - 85 data transfer sessions over 90 days; HTN - two BP and HR measures on an average of 453 out of 540 cumulative days *Patient acceptance* High, 17 patients continued with telemonitoring at study end. *Study completion/attrition:* 19 participants completed. One participant withdrew due to inability to operate the mobile phone because of poor vision. *Clinical utility* CHF patients: stable or improved: mean LVEF improved from 32% to 35%; Beta-blocker initiation supported: commenced and up titrated successfully in 10 of the 14 CHF patients Patients with HTN: BP stable; 134/80 mmHg at baseline vs 135/78 mmHg at completion
Scherr, 2009 Austria	Patients with heart failure and a hospital admission of > 24 h in the last 4 weeks. *n* = 120 (66 TMG; 54 SCG) TMG: *n* = 12 never beginners (50% M) TMG: *n* = 54 (74% M) CG: *n* = 54 (72% M) Metropolitan centres Rural patients not reported. Theoretical model: None	*mHealth* Mobile phone with Wireless Application Protocol (WAP) technology, Weight scale and automated BP monitor Secure web-based CRF at monitoring centre. Patients measured BP, HR and weight daily. Data entered and automatically sent to the remote server at remote Email alerts to study physician Study physician accessed data and could phone patient on mobile Study physician could set automatic reminders. *Non-mHealth* CG: Pharmacological care	Prospective, open-label RCT TMG: Pharmacological treatment with telemedical surveillance CG: pharmacological treatment alone and no planned interaction with study site. 6 months follow-up Long-term engagement: no engagement or follow-up reported beyond the 6 month intervention period. *Statistical analysis:* Per protocol principle and intention to treat analysis. Log-rank test, Kaplan-Meier estimation and relative risk reduction utilized for primary endpoint. Secondary endpoints: *t*-test, chi-square test, Wilcoxon rank sum test and Wilcoxon signed rank test utilized.	*Median age:* 12 never beginners 68 years (IQR 64–74); TMG 65 years (IQR 62–72); CG 67 years (IQR 61–72) *Study completion/attrition:* 104 participants completed. 12 participants were unable to transmit data - classified as never beginners. 4 TMG participants withdrew early (included in intention-to-treat and per-protocol analysis). *Participant adherence:* 95% *Intention-to-treat analysis:* TMG vs CG: 0 deaths and 11 hospitalizations (17%) vs 1 death and 17 hospitalizations (33%), a RRR of 50% (95% CI 3–74%), *p* = 0.06 TMG: majority of re-hospitalizations occurred in first month of follow-up. *Per protocol analysis:* TMG: 0 deaths and 8 hospitalizations (15%), a RRR of 54% (95%CI 7–79%), *p* = 0.04 NYHA class improved (III to II) in TMG, *P* < 0.001 vs CG and TMG baseline Median length of stay: TMG 6.5 vs SCG 10.0 days (IQR 7.0–13), p = 0.04 LVEF: ns improvement in both TMG and CG. TMG 25% (IQR 20–38) to 35% (IQR 25–45) and CG 29% (IQR 21–36) to 35% (IQR 24–40) 375 alerts, 170 contacts, 55 adjustments to heart failure medications.
Seto, 2012 Canada	Heart Failure patients with LVEF < 40% *n* = 100 TMG: *n* = 50 (82% M) SCG: *n* = 50 (76% M) Metropolitan centre (possible patients from rural or remote settings) Theoretical model: Self-care	*mHealth* *TMG:* Smartphone with blue tooth capability, BP monitor and scales; ECG recorder provided to 17 TMG participants – data automatically sent wirelessly to data repository. Daily morning symptom questions Email and text messages Website viewing of results by clinicians and patients. *Non-mHealth* *All* Pre and post study: Demographic, clinical data, SCHFI and MLHFQ questionnaires *SCG:* Clinic visits Optimization of medication Heart Failure education Telephone contact *TMG:* Standard care as per SCG	Non-blinded RCT TMG vs Standard Care Group (SCG) Stratified 4 block randomization based on NYHA classification. 6 month follow-up: post-study questionnaire; 22 semi-structured interviews with TMG participants; 5 semi-structured interviews with clinicians Long-term engagement: no engagement or follow-up reported beyond the 6 month intervention period. SCG participants were not contacted by the study site until study end. *Statistical analysis:* Between group analysis: Student t tests and Mann-Whitney tests. Within group analysis: Paired Student t tests and Wilcoxon signed rank tests.	*Mean age:* TMG 55.1 years (SD 13.7); SCG 52.3 years (13.7) *Completion/attrition:* 97 participants completed. 3 participants withdrew from TMG (1 was incapacitated after a fall; 2 because of technical difficulties). No participant withdrew from SCG. *Patient adherence:* 84%, 66% and 32% completed at least 50%, 80% and 95% of possible daily readings. *Health service utilization:* no significant differences in hospital admissions; nights in hospital; and ED visits. Number of Heart Function Clinic visits increased in TMG (*p* = 0.04) due to unplanned cardiologist recalls in response to telemonitoring system alerts. Improvement post-study for TMG and SCG - BNP values (*p* = 0.001 and *p* = 0.002); NYHA class (*p* = 0.000 and 0.001); LVEF (p = 0.001); and self-care (*p* = 0.004 and *P* = 0.006). QOL improved only in the TMG (*p* = 0.02). Between group post-study - only self-care maintenance (SCHFI) was significant (*p* = 0.03). Between group change - only overall QOL (MLHFQ) (*p* = 0.05)
Vuorinen, 2014, Finland	Heart Failure patients with LVEF ≤35%, NYHA class ≥2 n = 94 TMG: *n* = 47 (83% M) CG: n = 47 (83% M) Metropolitan centre (possible patients from rural or remote settings) Theoretical model: none	mHealth *Patient:* Mobile phone with preinstalled software app Provided with weight scale, blood pressure monitor, mobile phone with app and self-care instructions. Non-mHealth Multidisciplinary clinic visits and nurse feedback by telephone.	Prospective RCT TMG vs usual care (CG) 6-month follow-up Long term engagement: no engagement or follow-up reported beyond the 6-month intervention period *TMG:* Patients evaluated BP, HR, weight, symptoms and change in overall condition, weekly and transferred data via mobile phone application Received by secure remote patient monitoring server Patients received automated feedback about whether reported data was within personal targets set by nurse. *Nurses* accessed data and phoned patient weekly or as necessary for out-of-target parameters or failure to upload data *CG:* Patients encouraged to measure weight, blood pressure and heart rate at home Cardiac team monitor and interpret symptoms, optimize medication and provide education *Statistical analysis:* ZIP regression used for outcome variables that expressed counts, contiguous variables analysed within and between study groups	Mean age: TMG 58.3 (SD 11.6) CG 57.9 (11.9) *Completion/attrition:* 1 patient from TMG lost to follow-up. *Patient adherence =* proportion of weekly submitted self-measurements by TMG: 86% weight (median = 28 (IQR 23–33), 89% BP, HR, and symptoms (median BP and symptoms = 32 (IQR 27–43) *Feasibility and Acceptability: 96% (*44/46) from TMG responded to survey, 42/44 found making/reporting measurements with mobile app “useful” or “very useful.” 91% automatic feedback very or quite useful (9% no benefit), 66% feedback drew attention to essential issues of disease, 91% feedback was motivational. *Primary Outcome:* Mean HF-related hospital days: 0.7 (TMG) vs 1.4 (CG) (*p* = 0.351) *Secondary Outcomes:* *Clinical:* change in NT-proBNP, LVEF %, EHFSBS score, serum creatinine, potassium and sodium not significantly different between groups. Mortality: 0 (control), 0 (TMG). Within group changes were significant for: LVEF increased 5.0%, *p* = 0.003 TMG and 4.2%, *p* = 0.001 CG; EHFSBS (− 5.0 points, *p* < 0.001 TMG and − 3.8, p < 0.001); NT-proBNP decreased in the TMG (− 198 ng/l, *p* = 0.01) *Use of health care resources:* Mean nurse time, telephone contacts and visits higher in TMG (*p* < 0.001); TMG unplanned visits to Cardiac Outpatient Clinic higher (*p* < 0.001); TMG patient initiated telephone contact higher (*p* < 0.049); No statistical difference between groups for physician time and visits.

Key: *CHF* Chronic Heart Failure, *n/a* not applicable, *RCT* Randomised Controlled Trial, *TMG* Telemonitoring Group, *SCG* Standard Care Group, *CG* Control Group, *SCHFI* Self-Care of Heart Failure Index, *MLHFQ* Minnesota Living With Heart Failure, *NYHA* New York Heart Association, *LVEF* Left Ventricular Ejection Fraction, *BNP* Brain Natriuretic Peptide, *ED* Emergency Department, *QOL* Quality of Life, *ECG* Electrocardiogram, *BP* Blood Pressure, *HR* Heart Rate, *CV* Cardiovascular, *CRF* Case Report Form, *ED* Emergency Department, *NT-proBNP* N-terminal of the prohormone brain natriuretic peptide, *EHFSBS* European Heart Failure Self-Care Behaviour Scale

There was heterogeneity of the included studies related to study design, cardiac condition (ischaemic heart disease or heart failure) and outcome measures assessed. Levels of evidence, CASP score, theoretical framework and the differing outcome measures are reported in Tables 3 and 6 for CR and heart failure studies respectively. Outcomes are compared in Table 4 for CR and Table 7 for heart failure. CR and heart failure studies are summarised more fully in Tables 5 and 7 respectively.

### Cardiac rehabilitation

Of the five articles focusing on CR, three were feasibility, utility and uptake studies and two were RCTs (Tables 3 and 4). Worringham et al. was the only study to report the inclusion of rural participants [30]. Varnfield and colleagues utilized a model of self-management combined with the core components of a comprehensive CR program [31, 32].

### Feasibility, utility and uptake studies

A framework for the development and evaluation of mHealth has been developed by Whittaker and colleagues [33, 34]. Steps in this process include pretesting, feasibility and pilot studies to test the content, regimen and processes of the intervention, and outcome assessment to assess technical feasibility, process issues and the acceptability of the intervention to participants and staff [33, 34].

The three investigations of feasibility, utility and uptake of mHealth CR utilised differing study designs (Table 4) [30, 31, 35], with all three studies based around smartphones programmed with additional applications for exercise and/or education delivery and remotely monitored patient data. Data was synchronised to a server via a secure web portal, giving program staff the ability to assess participant outcomes and provide feedback in real-time [30, 31, 35]. Technology and patient-related measures are shown in Table 2 [30, 31, 35].

#### Feasibility

Delivering the core components of CR, either exercise alone or exercise and education via smartphone, was demonstrated to be technically feasible [30, 31, 35]. Participant engagement based on daily access was high [35], with usage [31] and session or task completions greater than 70% [30, 35], and ease of use was high (4.8/5) [30] (Tables 3 and 4). Qualitative feedback from participants and mentors [31] indicated an overall positive experience [35] and smartphone features were practical and easy to use [31] with a low frequency of minor technical problems [30]. However, Varnfield and colleagues reported that the Wellness Diary Connected internet portal was not regularly used by many participants (36%) due to lack of computer or internet access [31].

#### Uptake and utility

Uptake of mHealth CR was assessed through enrolment, engagement in, acceptance of and adherence to the program with all three studies reporting on at least three of these parameters. Overall, CR delivered by mHealth was well accepted, with good participant enrolment (86–93%) [30, 35], daily engagement (90%) [31, 35], and adherence and task completion rates (78–91%) [30, 31, 35] (Tables 3 and 4).

All three studies demonstrated mHealth effective for delivering the core components of CR [30, 31, 35]. Worringham et al. demonstrated significantly improved physical function (6MWT) and Quality of Life (QOL) (SF36 Physical Health Score) and a reduction in depression, although there was no significant change on the SF36 QOL mental health scale (Tables 3 and 4) [30]. Forman et al. reported that the Heart Coach application resulted in 42% lower visit cancellations and improved participant adherence. [35]. Cardiac Rehabilitation staff reported an overall positive impact on their ability to provide quality CR care by enabling them to better anticipate and address issues as they occurred. CR staff feedback included: “Allowed clinicians to connect with individuals who could not attend CR”; “increased educational class attendance”; “enhanced patient participation in CR activities and increased accountability in CR activities at home” (Tables 3 and 4) [35]. Varnfield et al. reported that both Care Assessment Platform (CAP) CR and phone consultations with mentors motivated participants to achieve their rehabilitation goals [31]. While mentors highlighted the benefits of CAP CR for patients, they expressed concerns over the lack of exercise supervision and group support (Tables 3 and 4) [31].

### Randomised controlled trials

Two RCTs identified are shown in Tables 3 and 4. Both studies were of similar study design; single-blind [36] or unblinded [32], parallel, two-arm RCTs. Blasco et al. also stratified by the presence of diabetes [36]. Follow-up periods were 6 and 12 months for the Varnfield and Blasco studies respectively.

#### Participants

Patients were included if they had a diagnosis of ACS and at least one coronary risk factor [36] or were post-Myocardial Infarction patients referred to CR (Tables 3 and 4) [32]. Participants were middle aged and the majority were male [32, 36]. Rural participants were not identified, and neither study reported on participant ethnicity or Indigenous status (Table 4) [32, 36].

#### Interventions

Two distinct intervention approaches have been utilized and are summarised in Table 4 [32, 36]. Blasco et al. utilized telemedicine as an adjunct to lifestyle counselling and usual care during a 12-month follow-up period with the aim of assessing the efficacy of the telemedicine system [36]. Varnfield and colleagues undertook a study aimed at examining whether CAP-CR was effective at improving CR use and health outcomes compared with traditional centre-based CR programs, and addressed all components of a comprehensive CR program via mHealth delivery in a RCT [32]. The CAP CR platform was downloaded onto a smartphone and provided health and exercise monitoring, delivery of motivational and educational information via text messages, and preinstalled audio and video files according to weekly themes [32].

#### Acceptability

The acceptability of mHealth CR was assessed by the number of sessions completed [36] or uptake, adherence and completion rates [32] and was demonstrated to be high in both studies (Tables 3 and 4). Varnfield et al. demonstrated significant increases in uptake, adherence and program completion compared with the TCR group (Tables 3 and 4). A small number of participant’s reported difficulty with using the mHealth tools [32] with low numbers of withdrawals occurring because of participant stress related to technology use or their inability to handle the technology [36] (Tables 3 and 4) .

#### Outcomes

The efficacy of CR delivered by mHealth (smartphone ± usual care and/or other eHealth methods) was as effective as or exceeded for some parameters that of traditional centre-based CR or usual care. Outcomes are shown in Tables 3 and 4 and outcome measures in Table 2.

On the basis of intention to treat analysis, Blasco et al. reported that the tele-monitoring group were significantly more likely compared with the control group (RR = 1.4; 95% CI = 1.1–1.7) to achieve the primary outcome of cardiovascular risk improvement (defined as the proportion of patients who achieved the goal of treatment in at least one cardiac risk factor without exacerbation of any of the others) and treatment goals for blood pressure (Tables 3 and 4) [36]. There was no significant between-group difference for smoking cessation or LDL-C. The tele-monitoring group achieved significant changes in all outcome measures (*p* < 0.05) with the exception of diastolic blood pressure; the control group achieved significant changes in diastolic blood pressure (*p* = 0.001) [36].

In the Varnfield et al. study, the primary outcomes of uptake, adherence and completion were 1.3, 1.4 and 1.7 times more likely in CAP-CR compared with traditional centre-based rehabilitation (TCR) [32]. Both CAP-CR and TCR were effective at improving the secondary outcomes from baseline to 6-week follow-up and between-group changes from baseline to 6-weeks were similar for both groups, with the exception of diastolic blood pressure and health related QOL (EQ5D)-index for CAP-CR and triglycerides for TCR [32] (Tables 3 and 4). An assessment of cost-effectiveness based on 2010 Australian health economics data suggested that increased CR completion rates with fewer admissions and deaths would result in AU$16.6 million readmission cost savings [32].

Table 3 reports a comparative summary of outcomes for the CR studies. Of the outcomes that were reported for both RCT studies, only quality of life was significantly improved in the mHealth interventions compared with control groups [32, 36]. Systolic and diastolic blood pressure, Haemoglobin A1_c_ and plasma lipid levels (LDL-c and triglycerides) were reported in both studies with inconsistent outcomes [32, 36].

### Heart failure studies

Four studies focused on improving outcomes through use of mHealth in heart failure rehabilitation and disease management: one feasibility, utility and uptake study, and three RCTs [37–39] (Tables 4, 5 and 7). Scherr et al. did not directly report on the inclusion of rural participants but did report poor reception of mobile phones in rural areas [37, 38]. Seto et al. included participants from metropolitan and possibly rural settings as indicated by the statement that some patients needed to travel a number of days prior to arriving home [39]. Vuorinen et al. reported that the study was conducted in a metropolitan area and that patients did not have to travel far to obtain health services [40]. Only Seto et al. utilised a theoretical framework, that of self-care, with measurement based on the Self-Care of Heart Failure Index (SCHFI) [39].

### Feasibility, utility and uptake study

Scherr et al. evaluated a newly developed telemedicine system for its acceptability, feasibility and reliability in supporting 14 patients (13 male) with heart failure and 6 (5 male) with hypertension in a 90 day observational study [37] (Tables 4, 5 and 7). Heart failure was defined as being symptomatic for at least six months, a mean left ventricular ejection fraction < 45%, a resting heart rate > 60 beats per minute and therapy with angiotensin-converting enzyme inhibitors. The telemedicine system integrated care through a mobile phone, a physician website via a personal computer and a server and participants were provided with an automatic blood pressure monitor and a digital weight scale for daily use. Data was entered into templates on the mobile phone and sent automatically to the server for monitoring by study physicians [37].

Overall, the reliability of server accessibility was high for both data transmission and website availability [37]. Poor access related to limited connectivity for mobile phones in rural areas accounted for unsuccessful data transmissions. The feasibility and acceptability of mHealth delivery for heart failure management was demonstrated. The level of implausible data entry was low and successful transmission, adherence with self-measurements and data entry were high over the 90 day monitoring period [37]. One dropout occurred due to an inability to operate the system because of low vision. Acceptability was high with only two reports of problems in reading the mobile phone display [37]. Data entry took approximately two minutes and was rated as acceptable. Patients also reported that electronic reminders improved their adherence to measurement and hence their awareness of body weight and blood pressure. Acceptability was further indicated by 17 patients continuing with monitoring after they completed the study [37].

The clinical status of patients with heart failure was stable or improved at study end, as indicated by mean left ventricular ejection fraction (LVEF). Telemonitoring also supported the initiation of beta-blocker therapy in patients with heart failure [37].

### Randomised controlled trials

Three randomised controlled trials were identified (Tables 4, 5 and 7), all open-label (non-blinded) trials where participants were randomly allocated to standard care plus telemonitoring (TMG) or standard care alone (SCG) [38–40]. All three interventions took place over six months without longer-term engagement or follow-up beyond the six-month intervention period.

#### Participants

All studies included patients with a diagnosis of heart failure [38, 39]. Scherr et al. included patients with worsening heart failure (acute cardiac decompensation) and a hospital admission lasting > 24 h within the previous 4 weeks [38]. Seto et al. included patients with a LVEF < 40% and an expected survival of greater than a year [39]. Vuorinen et al. included patients with LVEF ≤35%, and NYHA class of ≥2 [40]. Patients were middle aged (mean TMG 55 years and SCG 52 years and TMG 57.9 and SCG 58.3 years, in Seto et al. and Vuorinen et al. respectively) compared with an older patient group (median 66 years) in the study by Scherr et al. [38–40]. Although Seto et al. reported on ethnicity, Indigenous status was not identified [39].

#### Interventions

All studies utilized a telemedical (mobile phone with smartphone functionality or Wireless Application Protocol (WAP) technology) surveillance system to monitor patient status in addition to standard care compared with standard care alone [38, 39]. Scherr et al. compared pharmacological treatment and telemedical surveillance with pharmacological treatment alone. The combined primary endpoint was cardiovascular mortality or re-hospitalization for worsening heart failure [38]. Participants entered their daily measures and heart failure medication dosage into the mobile phone and sent them to the monitoring centre for review by study physicians. Email alerts were sent to the study physician if transmitted data was outside of individually adjustable parameters or if there was a weight increase of greater than 2 kg in 2 days. If necessary, study physicians contacted the patient using their mobile phone [38].

Seto et al. compared telemonitoring in addition to standard care with standard care alone [39] using brain natriuretic peptide (BNP), self-care and QOL as the primary outcome measures. The study was underpowered to detect between group differences in hospital readmissions, number of nights in hospital and mortality, and hence these were secondary outcomes [39]. Standard care consisted of visits to a Heart Function Clinic, medication optimisation, heart failure education and the ability to contact the clinic as necessary. The TMG utilised the telemonitoring system (phone, BP monitor, weight scales and ECG recorder) for daily monitoring and data was sent automatically via Bluetooth to the mobile phone and then to a data repository for review by clinicians and participants. Participant and clinician experience with the system was examined by semi-structured interviews [39].

Vuorinen et al. compared telemonitoring in addition to standard care against standard care alone [40]. Days spent in the hospital for heart failure was the primary outcome measure with multiple secondary outcomes including clinical outcomes, use of health care resources (mean time with nurse or physician, telephone contacts by nurse and by patient, visits to nurse, visits to physician, and unplanned visits to Cardiac Outpatient clinic) and patient experience [40]. Standard care consisted of self-measurement of HR, BP, and weight at home and regular visits to the cardiac clinic. Contact by telephone was added to standard care as necessary. The TMG utilized a mobile phone with a preinstalled software app for weekly monitoring of HR, BP, weight, and symptoms. Data was sent to a secure patient server where it could be accessed by the cardiac team through a web-based user interface. Participant experience with the system was elicited by survey [40].

#### Outcomes

Telemonitoring for heart failure management was demonstrated to be feasible and acceptable with high patient adherence and to the potential to reduce hospital service utilization through lowering the frequency and duration of hospitalisations [38, 39] (Outcome measures are shown in Table 4 and outcomes in Tables 5 and 7). Utilizing a per-protocol analysis, Scherr et al. demonstrated a significant 54% relative risk reduction (RRR) of hospitalisation for the telemonitoring compared with control group and although intention-to-treat analysis did not reach significance (RRR 50%, *p* = 0.06) [38]. However, the benefit of telemonitoring was not evident within the first month of follow-up when the majority of hospital re-admissions occurred. Compared with the controls, the per-protocol analysis demonstrated those with telemonitoring had a significantly shorter length of hospital stay for those hospitalised for worsening heart failure [38]. Seto et al. demonstrated an increased rate of cardiologist review for deteriorating health status identified through telemonitoring although no difference in hospital service utilisation; however, this was not a primary outcome and the study was underpowered to detect a difference in these parameters [39]. Vuorinen and colleagues demonstrated a non-significant reduction in heart failure-related hospital days in the telemonitoring group compared with the standard management (0.7 (SD 2.4) vs 1.4 (SD 3.5)). However, there was a significantly higher health care resource utilization in the telemonitoring group for mean nurse time, contacts and visits, cardiac outpatient clinic visits and patient initiated telephone contact but not physician time and visits [40].

In a per-protocol analysis, Scherr et al. demonstrated a significant improvement for New York Heart Association (NYHA) class and a non-significant improvement in left ventricular ejection fraction in those using telemonitoring [38]. While there were improvements demonstrated by Seto et al. in brain natriuretic peptide, NYHA class, LVEF, self-care maintenance and self-care management improved for both telemonitoring and standard management groups, physical and emotional QOL (measured by Minnesota Living with Heart Failure Questionnaire (MLHFQ)) were significantly improved for patients using telemonitoring [39]. Between-group post-study data analysis indicated that the group using telemonitoring had greater improvement in self-care maintenance and improvement in overall QOL (MLHFQ). Post-hoc subgroup analysis of 63 patients attending the Heart Failure clinic for greater than 6 months demonstrated significant improvement in BNP (*p* = 0.02), LVEF (*p* = 0.005), self-care maintenance (*p* = 0.05) and self-care management (*p* = 0.03)in the group using telemonitoring [39]. Vuorinen et al. demonstrated no significant improvements for NT-proBNP, LVEF, EHFSBS, and serum creatinine, potassium and sodium between groups [40]. However, in both groups there were significant improvements in LVEF and EHFSBS (*p* < 0.003) and a significant reduction in NT-proBNP with telemonitoring (*p* = 0.01). Medication adjustments, both increases and decreases, were significantly higher with telemonitoring compared with standard care [40].

## Discussion

Despite the heterogeneity of the studies reviewed, mHealth was shown to be feasible with high rates of participant engagement, acceptance, usage and adherence. The efficacy of mHealth was comparable to traditional centre-based CR, however, reductions in hospital service utilization for heart failure patients was inconsistent. mHealth has the potential to be an effective method of delivering CR and heart failure management and improving access for patients unable to attend traditional centre-based rehabilitation programs, however, larger high quality studies are required for more definitive conclusions to be drawn.

For smartphones to be an effective platform for supporting behaviour change and self-management of health conditions, the smartphone needs to be a vital and inseparable aspect of the intervention [41]. Intervention designs informed by behaviour change theory are more effective than those without a theoretical base [42]. A behaviour change framework, the Fogg Behaviour Model, provides for a shared understanding of human behaviour that is useful in the analysis and design of persuasive technologies [43]. The Fogg Behaviour Model identifies and defines three principle factors that control whether a behaviour is likely to be performed; motivation, ability and triggers [43]. Oinas-Kukkonen extended Fogg’s work by introducing a framework to classify technology in its persuasive functions, the persuasive system design [41, 44]. Persuasive systems are defined as “computerized software or information systems designed to reinforce, change or shape users’ attitudes or behaviours without using coercion or deception” [41]. Published studies of mHealth delivery of CR to date have not specifically addressed behaviour change strategies in intervention designs [45, 46]. However, a mHealth healthy-eating pilot study conducted by Dale et al. utilized a healthy eating intervention framed within social cognitive theory that resulted in a post-intervention increase in environmental self-efficacy when making food choices [47]. Behaviour change theories were limited to only 3 of 9 the studies in this review and included the theories of self-care [39] and a model of self-management [31, 32].

The development, evaluation and implementation of complex interventions and innovative approaches for the delivery of health care requires an iterative program of research with a systematic approach [33, 34, 48–50]. Both iterative components to explore discovery, development and evaluation of effectiveness leading to implementation and applied programmes of research for mHealth home-based CR or heart failure management have been reported [33, 34, 48, 49, 51]. Scherr and colleagues and Varnfield et al. show an iterative program of development, evaluation and implementation through feasibility studies and RCTs [31, 32, 37, 38].

A framework for developing and evaluating mobile applications for CR suggests the following six principles: the core components of CR should be addressed; individual tailoring of features are enabled; behaviour change theory is applied; high usability is demonstrated; patient centred outcomes are improved; and efficacy in a randomized clinical trial is established [45]. Varnfield et al. is the only identified RCT that comprehensively addresses the core components of CR via mHealth [32]. These are documented in the internal feasibility study (Table 1 in [31]) and in the RCT (Fig. 1 in [32]) where CR components were delivered by text messages, preinstalled audio and video files and mentoring sessions. Participants were also provided with the National Heart Foundation “My Heart My Life” manual [52].

Two design principles in the primary task support category of the persuasive system design framework are tailoring and personalization [41]. Both relate to the persuasive capabilities of the system and are closely related. Tailoring relates to the potential needs, interests, personality, usage context, or other factors relevant to a user group, whereas personalization relates to personalized content or services [41]. All studies included in this review had features that enabled personalization of information delivery, most commonly through feedback of results by telephone, telephone/video mentoring and individualised text messages. A study by Antypas found that tailoring a mobile phone intervention to enhance maintenance of physical activity after CR utilizing SMS text messaging resulted in no difference in perceptions of personal relevance of the intervention although compared with the control group the tailored intervention group maintained a significantly higher level of physical activity at 3 months post discharge [53]. In a systematic review of adherence to web-based interventions, Kelders et al. examined whether intervention characteristics and persuasive design affected adherence. They reported that primary task support plays a more important role in the effect of an intervention with differences in technology and interaction predicting adherence [44].

No RCT in this review included a follow-up of longer than 12 months. Out of the five RCT studies included, four studies had an intervention or follow-up period of 6 months [32, 38–40], while one had a 12 month follow-up period [36]. Given the chronic nature of the conditions these interventions address, the lack of long-term follow-up leaves open to question their longer term effectiveness. Patient feelings of abandonment and difficulties integrating into community exercise programs after CR have been reported [54], so how mHealth interventions fare warrants further study given the implications for the long-term maintenance.

Assessment of cost-effectiveness is an important component of pre-trial modelling [55] and evaluation of complex interventions [48, 56]. A cost-effectiveness analysis was not formally undertaken in any of the studies included in this review, although a cost-estimate based on 2010 Australian health economic data for CAP CR [32] found a likelihood of cost-savings based on higher CR completion rates and reduced hospital admissions and mortality [32]. Health service utilization was reported in the three heart failure RCT’s with inconsistent results. Significantly reduced hospitalisations and shorter length of hospital stay was reported in one study [38], whereas the other two studies reported no significant difference in heart failure related hospital days or admissions but a significant increase in Heart Function Clinic visits [39] or increased nurse time related to telephone contact (nurse and patient initiated) and unplanned clinic visits [40].

To date, evidence for mHealth effectiveness in CR or heart failure management has primarily included participants from metropolitan settings. There is a paucity of evidence for the adaptability and effectiveness of these programs in rural, remote and Indigenous patients, showing that further research is required [5]. Those studies reporting inclusion of rural patients [30, 37, 39] did not include subgroup analyses of outcomes for these patients, possibly due to a limited number of participants although periodic connectivity interruptions for rural participants were reported [30, 37]. This gap means that the feasibility of mHealth delivery of CR and heart failure management requires testing in rural and remote settings where non-centre based care is particularly needed. Implementation of mHealth in these settings requires robust evaluation.

This review included interventions utilizing smartphone functionality both through the use of a smartphone and through the use of mobile phones with Wireless Application Protocol (WAP) capabilities. Six studies used smartphones in their interventions, while three studies used mobile phones with WAP capabilities. While differences in outcomes for the two different types of smartphone functionality are not clear, the smartphone’s educational and other support capabilities should be considered.

Restriction of the literature search to smartphone functionality is a strength of this review in a nascent and fast growing method of health care delivery. The search excluded other alternative methods of home-based CR and heart failure management as these have been reported elsewhere [5]. The search also excluded mHealth interventions that solely used SMS text messaging. mHealth studies using SMS text-messaging were excluded because this approach does not comprehensively address the core components of CR, specifically exercise, education, and psychosocial support. SMS-based studies included interventions such as SMS goal setting and activity reminders and automated health promotion messages to promote exercise and smoking cessation [53, 57, 58], but they were limited in the amount of education, feedback, and psychosocial support they could provide. [53, 57, 58]. It is possible that utilizing a combination of multiple technology modalities (smartphones, SMS text messaging and/or mentoring by phone) may prove superior to the use of a single modality such as smartphone use alone. Indeed, Varnfield et al., highlighted the importance of mentoring interactions via the mobile phone to motivate patients to achieve their goals [31]. Limiting the search to smartphone functionality has resulted in a small number of studies being eligible for inclusion in this systematic review.

Another limitation of the studies was the relatively small sample size of some studies and the limited follow-up times of the RCTs available. Given the studies with smaller sample sizes (6 to 26 participants) were feasibility, utility and uptake studies [30, 31, 37], their inclusion does not impact on the analysis of effectiveness of mHealth compared with TCR in the RCTs reported on [32, 36, 38–40].

## Conclusion

mHealth delivery of CR and heart failure management is feasible with high rates of participant engagement, acceptance, usage and adherence. The efficacy of mHealth in these studies was comparable to traditional centre-based CR. mHealth delivery has the potential to improve access to CR and heart failure management for patients unable to attend traditional centre-based programs. The higher proportion of Indigenous people in more remote areas means that mHealth applications for particular subgroups needs special consideration. Feasibility testing of mHealth delivery for CR and heart failure management for rural and remote settings in Australia should include assessment of cultural compatibility with careful evaluation of implementation for rurally based health services and consumers.

## Additional file

Additional file 1: Search Strategies. This supplementary document details four database search strategies: Three for all databases searched except Medline and the fourth is for MEDLINE. (DOC 34 kb)

## Abbreviations

ACS: Acute Coronary Syndrome
BNP: Brain Natriuretic Peptide
CAP: Care Assessment Platform
CASP: Critical Appraisal Skills Programme
CHF: Chronic Heart Failure
CR: Cardiac rehabilitation
CVD: Cardiovascular disease
DBP: Diastolic Blood Pressure
ECG: Electrocardiogram
ED: Emergency Department
FUU: Feasibility, Utility and Uptake study
GPS: Global positioning system
HbA1_c_: Haemoglobin A1_c_
HTN: Hypertension
ICT: Information and communication technologies
LVEF: Left ventricular ejection fraction
mHealth: Mobile health
MLHFQ: Minnesota Living with Heart Failure Questionnaire
NHMRC: National Health and Medical Research Council
NYHA: New York Heart Association
PDAs: Personal digital assistants
QOL: Quality of Life
RCT: Randomised Control Trial
SBP: Systolic Blood Pressure
SCG: Standard care alone group
SMS: Short messaging service
TMG: Telemonitoring group
